# Insect wing tarsal foreign body causing conjunctival granuloma and marginal keratitis

**DOI:** 10.4103/0301-4738.57154

**Published:** 2009

**Authors:** Kalpana Babu, Rashmi E Y Maralihalli

**Affiliations:** Vittala International Institute of Ophthalmology & Prabha Eye Clinic and Research Center, Bangalore, India

**Keywords:** Conjunctival granuloma, insect wing foreign body, marginal keratitis

## Abstract

A 37 year old male was referred to our centre for management of episcleritis with peripheral keratitis in the right eye. He had a history of ocular discomfort in the right eye of 1 week duration. Slitlamp examination revealed marginal keratitis between 12'o clock to 2'o clock positions in the right eye. Lid eversion revealed an insect wing on the tarsal conjunctiva along with an adjacent conjunctival granuloma. The area of the marginal keratitis corresponded to the area of the foreign body and the conjunctival granuloma. The probable mechanism of the development of marginal keratitis and the conjunctiva granuloma is speculated in this case report.

We report an interesting case of an insect wing on the right superior tarsal conjunctiva causing a localized conjunctival granuloma and marginal keratitis with pannus.

## Case Report

**Figure 1:**
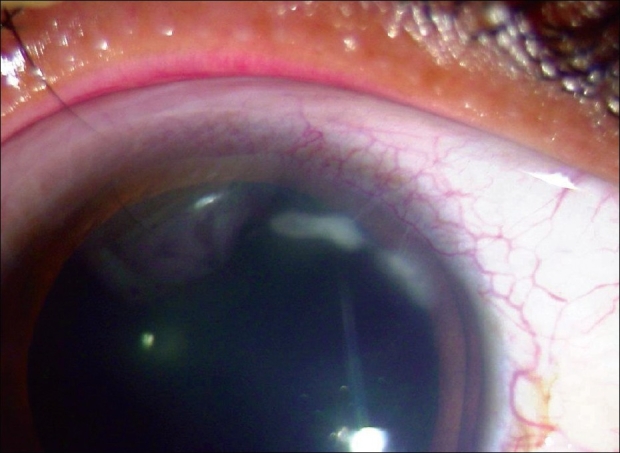
Slit-lamp photograph of the right eye with marginal keratitis between 12'0 clock to 2'0 clock positions along with a pannus

**Figure 2:**
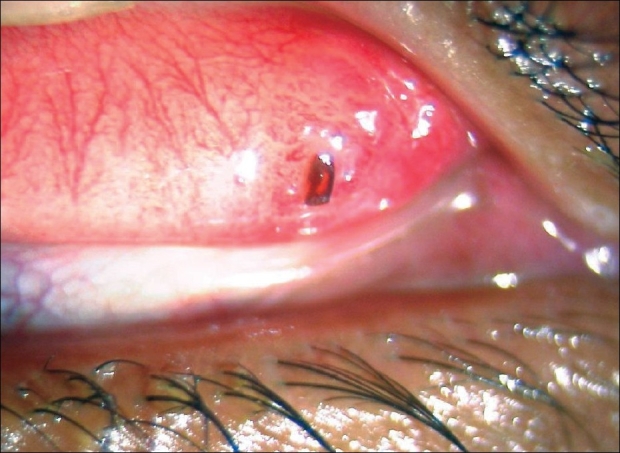
Slit-lamp photograph of the insect wing foreign body on the tarsal conjunctiva causing a localized conjunctival granuloma

A 37-year-old male was referred with a diagnosis of episcleritis with peripheral keratitis to our center for further management. He had a history of increasing ocular discomfort in the right eye of one week duration. He was not on any medicines at the time of presentation. On examination, his best-corrected visual acuity was 20/20 in both eyes. Slit-lamp examination in the right eye revealed marginal keratitis between 12'0 clock to 2'0 clock positions along with a pannus [Fig. 1]. Eversion of the eyelid revealed an insect wing on the superior tarsus with a localized conjunctival granuloma at the area of the foreign body [Fig. 2]. The lid margins were healthy with no evidence of blepharitis. The marginal keratitis corresponded to the area of the foreign body and the conjunctival granuloma. Rest of the ocular examination was normal. Left eye examination was normal. The foreign body was removed under topical anesthesia with a forceps and sent to a microbiologist for confirmation. Confocal microscopy (Rostock corneal Module II, Heidelberg retinal tomogram, Heidelberg, Germany) at the area of the keratitis and corneal scraping (KOH, Grams stain and culture) were negative for an infectious etiology. Topical treatment initially with antibiotics (ofloxacin, four times a day for a week) and lubricants (1% carboxy methyl cellulose, six times a day) followed by a course of steroids started three days later (dexamethasone, four times a day for five days) and tapered over three weeks alleviated his symptoms and caused resolution of the conjunctival granuloma over four weeks.

Our case highlights the need for a complete ocular examination in every case of keratitis or scleritis. Insect wing foreign bodies on the cornea and the peripheral limbus causing vascularization, infiltration and secondary bacterial infections have been reported.[1] There is only an isolated report of an insect wing tarsal foreign body causing a localized conjunctival granuloma.[2] However, there are no reports of marginal keratitis associated with an insect wing tarsal foreign body (Medline search). When a foreign body gets lodged in the conjunctiva, initially, there is an acute inflammatory response in the form of exudation of plasma and fibrin. This normally dislodges the foreign body. However, when the foreign body has a large surface area, as in this case, this mechanism may be insufficient and the foreign body becomes embedded. This is followed by a chronic inflammatory response resulting in the formation of a granuloma containing epitheloid and foreign body giant cells.[2,3] The presence of the foreign body and the exotoxins released by the bacterial flora around the foreign body may have resulted in sterile infiltrates (marginal keratitis) in the cornea. It is also possible that the corneal findings were secondary to the abrasive effect of the foreign body. Any sustained paralimbal inflammation evokes a fibrovascular proliferation in the form of a pannus as seen in our case.
